# Invasive Mucinous Adenocarcinoma of the Lung Presenting With Multiple Cavities

**DOI:** 10.7759/cureus.13795

**Published:** 2021-03-10

**Authors:** Shunsuke Nakamura, Hiroshi Sugimoto, Kazuki Negoro, Ryuichiro Tanaka

**Affiliations:** 1 Department of Respiratory Medicine, Kobe Red Cross Hospital, Kobe, JPN

**Keywords:** invasive mucinous adenocarcinoma, lung cancer, lung cavity

## Abstract

A 78-year-old woman presented to our hospital with a two-week history of productive cough. Chest computed tomography (CT) showed bilateral multiple pulmonary nodules with cavities. Although the cytology of her sputum revealed adenocarcinoma, she refused any treatment. Following supportive care, 30 months later, she presented to our hospital with dyspnea and fever. Chest CT showed progression of multiple pulmonary nodules and cavities. Despite treatment with antibiotics and palliative care, she died on the 10th day of hospitalization. Pathological autopsy confirmed the diagnosis of pulmonary invasive mucinous adenocarcinoma (IMA). The typical CT findings of IMA include multiple consolidations or ground-glass opacities mimicking pneumonia; rarely, cavitary lesions are also observed. Clinicians should consider IMA as a differential diagnosis for lung cavities.

## Introduction

Invasive mucinous adenocarcinoma (IMA), formerly classified as mucinous bronchioloalveolar carcinoma, is a variant of adenocarcinoma according to the International Association for the Study of Lung Cancer/American Thoracic Society/European Respiratory Society lung adenocarcinoma classification system [1]. The typical computed tomography (CT) finding of IMA is multiple consolidations or ground-glass opacities mimicking pneumonia; however, there are a few reports of IMA with cavitary lesions [2,3]. Here, we report a case of IMA with multiple lung cavities.

## Case presentation

**Figure 1:**
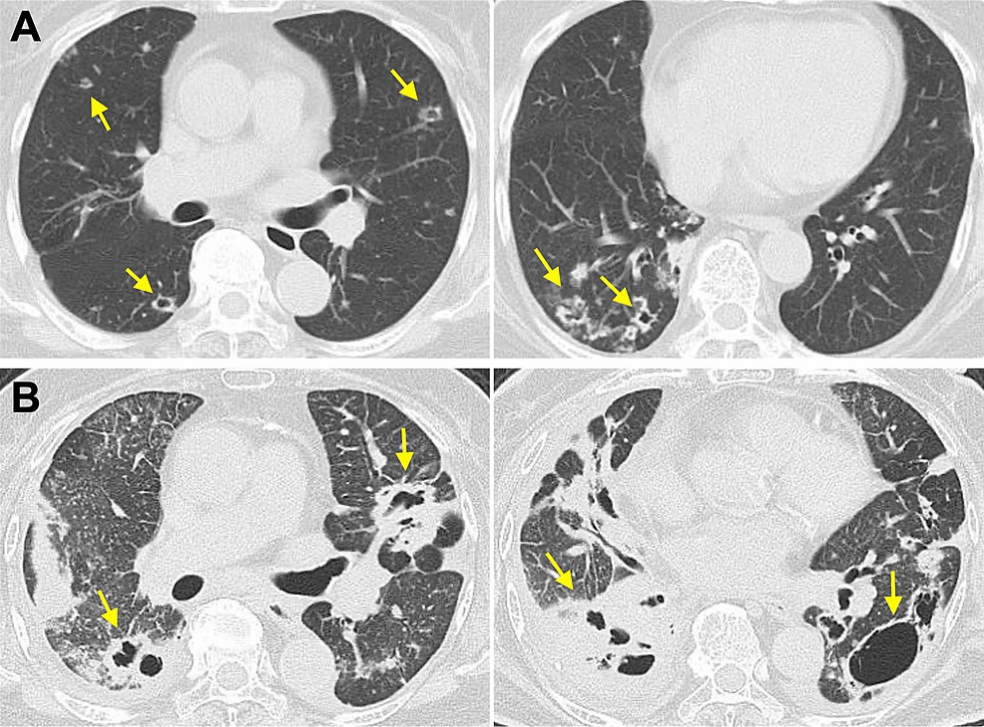
Chest CT findings. (A) Multiple pulmonary nodules with cavities (arrows). (B) The progression of multiple pulmonary nodules with cavities (arrows), thickening of interlobular septa and bronchovascular bundles, and bilateral pleural effusions, 13 months later. CT, computed tomography

A 78-year-old Japanese woman presented to our hospital with a two-week history of productive cough with no history of cigarette smoking. She had no notable medical history except for uncontrolled diabetes mellitus. Chest CT showed multiple bilateral pulmonary nodules with cavities (Figure 1A). The cytology of her sputum revealed adenocarcinoma. There was no extrapulmonary metastasis. We recommended chemotherapy for lung cancer, but she refused any treatment.

Thirty months later, she presented to our hospital with dyspnea and fever. Her vital signs were as follows: body temperature, 37.5°C; blood pressure, 130/90 mmHg; heart rate, 125/min; and peripheral oxygen saturation, 95% with 2 L/min of oxygen via nasal cannula. Chest CT showed multiple pulmonary nodules and cavities, thickening of interlobular septa and bronchovascular bundles, and bilateral pleural effusions (Figure 1B). The results of transthoracic echocardiography were within the normal range. Blood tests showed an elevated white blood cell count of 17,900/μL (normal, 3,900-9,800/μL), C-reactive protein level of 14.02 mg/dL (normal, 0-0.3 mg/dL), and B-type natriuretic peptide of 16.8 pg/mL (normal, 0-18.5 pg/mL). All culture results (blood, urine, and sputum) were negative.

**Figure 2:**
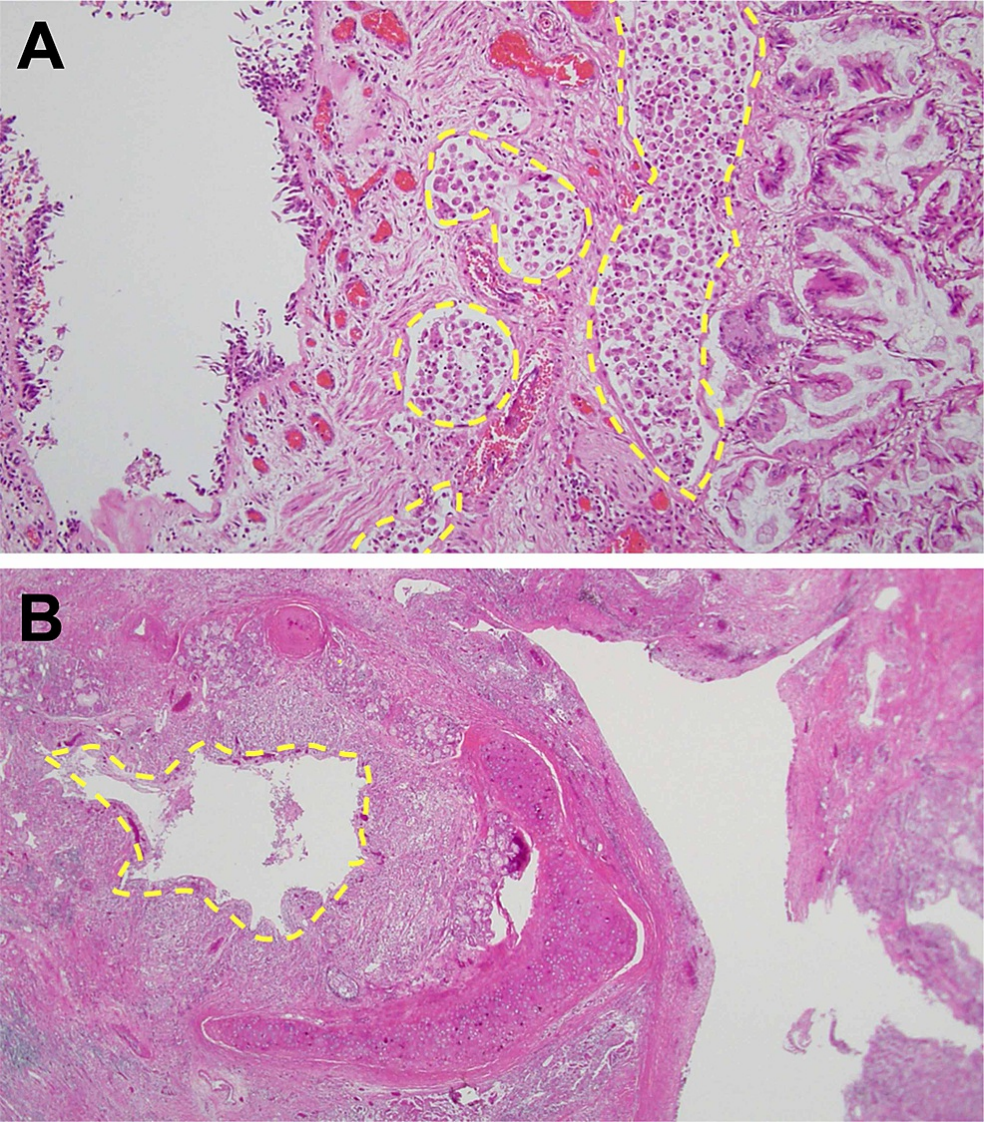
Pathological autopsy findings. (A) IMA with columnar malignant cells containing abundant mucin, invading into lymphatic vessels (dotted lines). (B) Bronchial stenosis due to invasion of malignant cells (dotted lines). IMA, invasive mucinous adenocarcinoma

Despite treatment with antibiotics and palliative care, she gradually deteriorated and died on the 10th day. We confirmed the diagnosis of pulmonary lymphangitic carcinomatosis due to pulmonary IMA, following pathological autopsy (Figure 2A). Microscopic findings of the autopsied lung showed columnar malignant cells containing abundant mucin, which is consistent with IMA and bronchial stenoses due to invasion of malignant cells (Figure 2B).

## Discussion

IMA is a rare variant of adenocarcinoma, accounting for approximately 5-10% of lung adenocarcinomas [1,4]. IMA is often located in the lower lobe of the lung and tends to spread along the airway; thus, IMA readily metastasizes to other lung lobes including the contralateral lung. Furthermore, it has been reported that disease-free survival at five years is 76% among patients with IMA [5].

Typical CT findings of IMA include multiple consolidations or ground-glass opacities mimicking pneumonia; however, there are some reports that a solitary nodule pattern is much more common than pneumonia pattern [2,6]. Pathological features of IMA are characterized by goblet or columnar tumor cells containing abundant cytoplasmic mucin, and a lepidic growth pattern with microscopic skip lesions [7].

In the present case, pneumonia-like CT findings in the right lower lobe were consistent with the features of IMA, whereas the presence of multiple cavities was unusual. Multiple cavitary lesions in the lung can be observed in many diseases such as tuberculosis, nontuberculous mycobacterial infection, and primary or metastatic lung cancer [8]. To the best of our knowledge, there are a few case reports of IMAs with cavities [2,3]. It has been reported that 7-13% of IMAs are accompanied by a cavity, and cavitary IMA tends to have a poor prognosis compared with noncavitary IMA [9,10].

Various mechanisms have been suggested for cavity formation in IMA; necrosis due to poor blood supply, release of proteolytic enzymes from the tumor cells, destruction of alveolar walls by excessive mucus, and tumor invasion into preexisting lung cysts [2]. In our case, we assumed that the mechanism of cavitary formation was check-valve formation because normal bronchial epithelial cells remained in the cavities and there were no preexisting lung cysts.

## Conclusions

We reported a case of cavitary IMA, which is a rare variant of adenocarcinoma. Although IMA is a relatively rare cause of cavitary lesions, cavitary IMA tends to have a poor prognosis compared with noncavitary IMA. Therefore, clinicians should keep in mind IMA as a differential diagnosis for lung cavities for the prompt diagnosis and appropriate treatment.

## References

[1] Travis WD, Brambilla E, Noguchi M (2011). International Association for the Study of Lung Cancer/American Thoracic Society/European Respiratory Society international multidisciplinary classification of lung adenocarcinoma. J Thorac Oncol.

[2] Masuzawa K, Minematsu N, Sasaki M (2017). Invasive mucinous adenocarcinoma of the lung presenting as a large, thin-walled cyst: a case report and literature review. Mol Clin Oncol.

[3] Verma R, Bhalla AS, Goyal A, Jain D, Loganathan N, Guleria R (2017). Ominous lung cavity "Tambourine sign". World J Clin Cases.

[4] Guo M, Tomoshige K, Meister M (2017). Gene signature driving invasive mucinous adenocarcinoma of the lung. EMBO Mol Med.

[5] Yoshizawa A, Motoi N, Riely GJ (2011). Impact of proposed IASLC/ATS/ERS classification of lung adenocarcinoma: prognostic subgroups and implications for further revision of staging based on analysis of 514 stage I cases. Mod Pathol.

[6] Cha MJ, Lee KS, Kim TJ (2019). Solitary nodular invasive mucinous adenocarcinoma of the lung: imaging diagnosis using the morphologic-metabolic dissociation sign. Korean J Radiol.

[7] Cha YJ, Shim HS (2017). Biology of invasive mucinous adenocarcinoma of the lung. Transl Lung Cancer Res.

[8] Ryu JH, Swensen SJ (2003). Cystic and cavitary lung diseases: focal and diffuse. Mayo Clin Proc.

[9] Jung JI, Kim H, Park SH (2001). CT differentiation of pneumonic-type bronchioloalveolar cell carcinoma and infectious pneumonia. Br J Radiol.

[10] Wang T, Yang Y, Liu X (2020). Primary invasive mucinous adenocarcinoma of the lung: prognostic value of CT imaging features combined with clinical factors. Korean J Radiol.

